# Pervasive Adaptive Evolution in Primate Seminal Proteins 

**DOI:** 10.1371/journal.pgen.0010035

**Published:** 2005-09-16

**Authors:** Nathaniel L Clark, Willie J Swanson

**Affiliations:** Department of Genome Sciences, University of Washington, Seattle, Washington, United States of America; National Institute of Genetics, Japan

## Abstract

Seminal fluid proteins show striking effects on reproduction, involving manipulation of female behavior and physiology, mechanisms of sperm competition, and pathogen defense. Strong adaptive pressures are expected for such manifestations of sexual selection and host defense, but the extent of positive selection in seminal fluid proteins from divergent taxa is unknown. We identified adaptive evolution in primate seminal proteins using genomic resources in a tissue-specific study. We found extensive signatures of positive selection when comparing 161 human seminal fluid proteins and 2,858 prostate-expressed genes to those in chimpanzee. Seven of eight outstanding genes yielded statistically significant evidence of positive selection when analyzed in divergent primates. Functional clues were gained through divergent analysis, including several cases of species-specific loss of function in copulatory plug genes, and statistically significant spatial clustering of positively selected sites near the active site of kallikrein 2. This study reveals previously unidentified positive selection in seven primate seminal proteins, and when considered with findings in *Drosophila,* indicates that extensive positive selection is found in seminal fluid across divergent taxonomic groups.

## Synopsis

Proteins found in seminal fluid accompanying sperm show dramatic effects on reproduction, such as manipulating female behavior. Even in primates they participate in competition between sperm of different males, and serve to protect sperm from infection by pathogens. These types of roles require the proteins to constantly adapt to stay ahead of the competition. Such adaptive pressures on proteins leave characteristic signatures in the DNA sequences that encode them. The authors used these signatures to identify adaptive evolution in primate seminal proteins and found extensive signs of adaptation when comparing thousands of seminal genes between human and chimpanzee. They further characterized outstanding genes in several primate species, including a diversity of apes and monkeys. Several of these proteins have no known function, yet by visualizing the adaptation on their three-dimensional surfaces, the authors uncovered clues to what is driving their evolution. In addition, they found several cases in which certain species lost their functional copies of these genes. Interestingly, species that showed loss of function do not participate in sperm competition. Past studies found widespread adaptation in fruit fly seminal fluid, and this study reveals extensive adaptation in primate seminal proteins. Could this be a phenomenon common among animals?

## Introduction

Studies of adaptive evolution have revealed multiple classes of reproductive proteins under positive selection, including those involved in gamete recognition, seminal fluid factors, and proteins in the female reproductive tract [1–5]. The unknown pressures driving this adaptive evolution may be shared among taxonomic groups. For example, evidence of positive selection in gamete recognition proteins is found across divergent taxonomic groups, including mollusks, echinoderms, green algae, and mammals [1,4–7]. Positive selection in seminal proteins is observed in *Drosophila* and in primate semenogelin proteins [2,8–10]. However, the extent of selection in primates remains unknown, and it has not been determined whether seminal fluid proteins in divergent taxa experience such adaptive pressures.

Seminal fluid proteins in *Drosophila* initiate striking reproductive responses in females [11]. Inseminated proteins have been shown to affect sperm storage in the female reproductive tract, copulatory plug formation, ovulation, oogenesis, female receptivity to re-mating, and female lifespan. These can be important effects for sperm competition and sexual conflict, both of which may drive adaptive evolution. Additional seminal factors show antibacterial activity and may serve in pathogen defense, another adaptive driving force.

There is reason to believe that similar forces act on primate seminal fluid, leaving signatures of positive selection. As in drosophilids, several primate species form a post-mating copulatory plug, which could serve in sperm competition by excluding subsequent ejaculates from competing males. Plugs are present in diverse primates [12], including prosimians, New World and Old World monkeys, and in the chimpanzee, the closest living relative to humans. Consistent with adaptation, positive selection is seen in semenogelin proteins functioning in this pathway [9,10,13].

To identify positive selection in primate proteins, we used a measure of selective pressure, the *d*_N_/*d*_S_ ratio [14,15]. Positive selection for amino acid diversification results in the rate of nonsynonymous substitutions exceeding that of synonymous substitutions. This effect is measured on coding sequences as the nonsynonymous substitution rate divided by the synonymous substitution rate— the *d*_N_/*d*_S_ ratio. A value greater than one is indicative of positive selection, and a value less than one indicates purifying selection. In the absence of selection (neutral evolution), a value of one is expected. When measured over the entire length of a gene, the *d*_N_/*d*_S_ ratio is a conservative measure of positive selection; in the presence of strong positive selection at some sites, conservation at others will lower the ratio. For this reason, when measured over the entire gene, we consider an elevated *d*_N_/*d*_S_ to be suggestive of positive selection acting on a portion of the gene [3]. We then measure statistical significance using multiple species alignments through a likelihood method (CODEML) allowing different *d*_N_/*d*_S_ values for codon sites [5,16,17]. Criticisms of this method include the argument that it gives false positive results under certain parameter combinations [18]. A more extensive study conducted by Wong et al. [19] found that this problem was limited to an early version of the program and to problems with convergence, and that the maximum likelihood method has good power and accuracy in detecting positive selection.

In this study we aimed to determine the extent of positive selection in primate seminal fluid proteins and to characterize outstanding candidates in further detail. Eight candidates were chosen from a pairwise human-chimpanzee *d*_N_/*d*_S_ screen of seminal proteins. More detailed analysis with several species sequences provided strong evidence that positive selection acts on several primate seminal proteins.

## Results

### A Selective Pressure Screen

A list of proteins present in human seminal fluid was compiled from mass spectrometry studies of seminal plasma and prostasomes [20,21]. A total of 161 proteins were identified, 129 in prostasomes and 43 in seminal plasma, with 11 found in both studies. Human coding regions for these genes were aligned with chimpanzee orthologous sequences in order to estimate selective pressure between these two lineages as indicated by the *d*_N_/*d*_S_ ratio. Estimates of pairwise *d*_N_/*d*_S_ ratios were calculated using both the Nei and Gojobori method and a maximum likelihood method [14,16]. Both gave similar estimates (Table S1).

**Figure 1:**
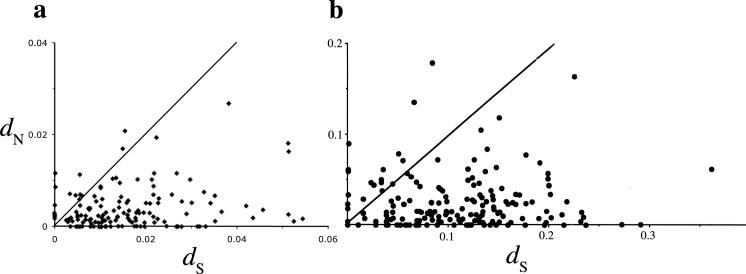
Plots of *d*_N_ Versus *d*_S_ for Primate and *Drosophila* Seminal Fluid Genes (A) Genes encoding seminal fluid proteins identified by mass spectrometry in human versus chimpanzee. (B) *Drosophila simulans* male-specific accessory gland genes versus *D. melanogaster* [2]. The diagonal represents neutral evolution, a *d*_N_/*d*_S_ ratio of one. Most genes are subject to purifying selection and fall below the diagonal, while several genes fall above or near the line suggesting positive selection. Comparison of the two plots shows elevated *d*_N_/*d*_S_ ratios in seminal fluid genes of both taxonomic groups.

Rates of nonsynonymous versus synonymous substitution in these genes revealed several coding sequences with elevated *d*_N_/*d*_S_ ratios (Figure 1A). Of 161 seminal fluid proteins, 17 had a *d*_N_/*d*_S_ greater than one, and 36 greater than 0.5. The median *d*_N_/*d*_S_ value was 0.19. A study of *Drosophila* seminal proteins showed similar variation of selective pressure (Figure 1B) [2]. Primate genes with elevated *d*_N_/*d*_S_ ratios were involved in immune response *(complement component 7, interleukin 1 receptor-like 2),* semen coagulum *(semenogelins I* and *II, prostate-specific transglutaminase 4, prostatic acid phosphatase),* cellular structure *(desmoglein 1, profilin I),* and other roles, including several proteins of unknown function. Results for all 161 genes are shown in Table S1.

Secreted proteins may tend to have higher *d*_N_/*d*_S_ values, as they encounter adaptive pressures from exterior forces, such as interactions in the female reproductive tract. The subset of 43 proteins with secretion signal sequences showed a higher mean *d*_N_/*d*_S_ (0.30) than those without (0.15). This difference is significant as determined by permutations (*p* = 0.0091). This 2-fold increase for secreted proteins was also observed in *Drosophila* seminal fluid [2].

Since the mass spectrometry studies are not expected to provide an exhaustive catalog of seminal fluid proteins, a similar screen was performed on prostate-expressed genes identified from an expression study of noncancerous human prostate [22]. Of 2,858 prostate-expressed genes, 290 showed a *d*_N_/*d*_S_ greater than one, while the median value was 0.15. Secreted proteins again showed a higher mean *d*_N_/*d*_S_ (0.24 versus 0.17, *p* = 0.00038).

The pairwise estimates were aimed at predicting candidate genes under selection, and eight were selected for in-depth analysis. Criteria used to select these candidates were a high *d*_N_/*d*_S_ value, a high *d*_N_, and evidence for high or specific prostate expression. Seven of eight candidates were taken from the mass spectrometry set because of the direct evidence that they are present in ejaculate. One gene, *kallikrein 2 (KLK2),* was taken from the prostate-expressed set, since it has a known role in seminal fluid dynamics. An additional gene, *prostate-specific antigen (PSA),* was analyzed due to its importance in copulatory plug dissolution, despite its low pairwise *d*_N_/*d*_S_ value (Table 1). Since it was not chosen from the screen results, *PSA* is not considered a candidate. Overall, we chose three candidate genes involved in semen coagulation and five candidates whose functions are unknown.

**Table 1:**
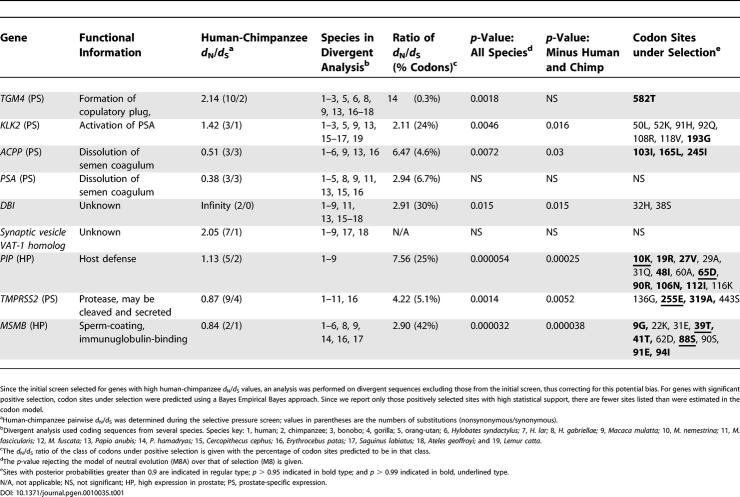
Seven Candidate Genes from the Screen Show Signs of Positive Selection

### Positive Selection in Candidate Genes

To assess statistical significance of positive selection in candidate genes, we sequenced primate coding regions to provide eleven species sequences on average. We then assessed the selective pressure acting on these sequences using *d*_N_/*d*_S_ ratios. Using a method that predicts a uniform *d*_N_/*d*_S_ ratio across all codon sites, several pairwise comparisons of *prolactin-induced protein (PIP)* and *β*-*microseminoprotein (MSMB)* sequences have *d*_N_/*d*_S_ ratios significantly greater than one, suggesting positive selection (unpublished data) [23]. This is a conservative approach, since it is unlikely that all codon sites are subject to the same selective pressure during evolution. More sensitive methods allow testing for variation in *d*_N_/*d*_S_ at codon sites by comparing neutral models to selection models of codon evolution. Model parameters were estimated using a maximum likelihood method employed in the CODEML program of the PAML package [5,16,17]. For each gene, three different comparisons of neutral and selection models gave similar results (M1 versus M2, M7 versus M8, and M8A versus M8). From these comparisons, significant signs of positive selection were found in seven of eight candidate genes (Table 1). Since candidates were chosen based on high human-chimpanzee *d*_N_/*d*_S_ values, there could be a statistical bias when sequences from the initial screen are included in the multiple alignments. When human and chimpanzee sequences were removed, six of the seven remained statistically significant, showing positive selection. The analysis that failed this conservative test, that of *prostate-specific transglutaminase 4 (TGM4),* may have suffered a lack of power, because the total tree length (0.47) was below optimal (~1) for this maximum likelihood method, due to the removal of two taxonomic groups [24].

The codon classes predicted to be under positive selection had *d*_N_/*d*_S_ values ranging from 2 to 14 and were estimated to contain large proportions of codons for some genes *(MSMB, PIP)* and smaller proportions for others *(TGM4)* (Table 1). The rapid evolution of *MSMB* was noted in past studies of primate, rodent, and bird sequences [25,26], and was attributed to either low selective constraint or positive selection. We found highly significant signs of positive selection within primates (*p* < 0.001), with an estimated 42% of codons showing a *d*_N_/*d*_S_ ratio of 2.90. Three diversified paralogs of the *MSMB* gene exist in New World monkeys [27], and their functions are unknown. When only Old World monkey and ape sequences are analyzed, significant positive selection is still observed (*p* = 0.029), and selection is predicted at similar codon sites.

**Figure 2:**
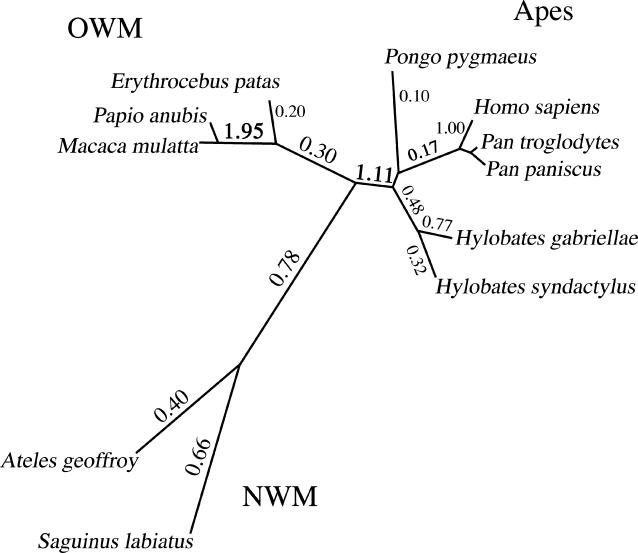
Variable Selective Pressure is Seen Between Lineages for Semen Coagulum Protein TGM4 This primate phylogeny shows selective pressure on *TGM4* with estimated *d*_N_/*d*_S_ ratios indicated on branches. Ratios greater than one are suggestive of either relaxed constraint or positive selection. Ratios are only shown for long branches, those with at least eight substitutions. A null model with a uniform *d*_N_/*d*_S_ ratio across all lineages is rejected in favor of these estimates (*p* = 0.003). Branch lengths are estimated from *TGM4* coding sequences. NWM, New World monkeys; OWM, Old World monkeys.

We looked for lineage-specific variation in selective pressure by estimating *d*_N_/*d*_S_ along phylogenetic lineages. For *TGM4,* a model estimating independent *d*_N_/*d*_S_ ratios for each lineage fit the data better than a model with a uniform ratio (*p* = 0.0031). This indicates that variable selective pressure acted on *TGM4* during its evolution, with branch-specific *d*_N_/*d*_S_ values ranging from 0.1 to 1.95 (Figure 2). *Prostatic acid phosphatase (ACPP)* also shows significant variation in *d*_N_/*d*_S_, with elevated values in the chimpanzee and rhesus macaque lineages—1.16 and 0.64, respectively (*p* = 0.016). Finally, *PSA* does not have a high pairwise human-chimpanzee *d*_N_/*d*_S_, but it shows significant variation in selective pressure during its evolution. A branch model shows *PSA* lineages with *d*_N_/*d*_S_ ratios exceeding one and was a significantly better fit than a model with uniform ratios for all lineages (*p* = 0.004). The extreme values in all three of these genes could be due to either positive selection or a reduction in functional constraint.

### Spatial Distributions of Selected Sites on Three-Dimensional Structures

Positively-selected codon sites were predicted by a Bayes Empirical Bayes method for all genes showing significant positive selection [28]. Observed levels of divergence and number of sequences were appropriate for accurate prediction of sites according to a power analysis of Bayes prediction [29]. The spatial relationship of these selected sites was evaluated by mapping them onto three-dimensional protein structures. This analysis was done to find connections between positive selection and functional sites, because previous studies of MHC, lysin, and ZP3 proteins showed that predicted sites of positive selection fall into regions or binding clefts where diversification is biologically relevant [4,5]. We mapped selected sites onto five primate seminal proteins employing either solved crystal structures or threaded structural models, and used only predicted sites with a high level of support (*p* > 0.9). The spatial patterns and locations yielded intriguing patterns of positive selection.

**Figure 3:**
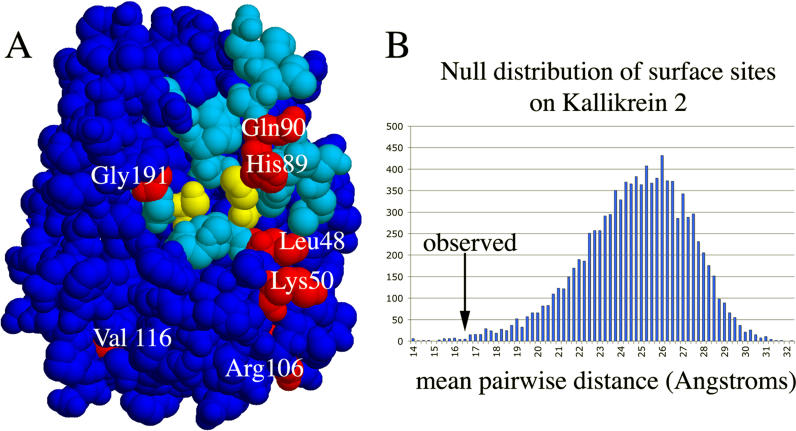
Positive Selection at Sites Involved in Substrate Binding in KLK2 (A) Several amino acid sites predicted to be under positive selection (red) are near the protease active site (yellow). Three selected sites are found in known structural components of kallikrein proteins (light blue residues): Gly191 is part of the S1 substrate binding pocket, and His89 and Gln90 are part of the kallikrein loop [30]. Selected sites are labeled with the human residue on this threaded model. (B) Positively selected sites are significantly clustered on the surface of KLK2. The observed mean pairwise distance between predicted positively selected sites is significantly lower than random sets of surface sites (*p* = 0.0043). This spatial clustering suggests that positive selection acted during KLK2 evolution to alter substrate binding.

Positively selected sites of the KLK2 protein fall near the active site residues and are found in known functional regions (Figure 3A). One selected site is in a known substrate binding cleft and two are in the kallikrein loop [30]. These locations and the pattern of clustering suggest that there was selective pressure for KLK2 to change substrate binding affinity. To assess statistical significance of surface clustering, we compared the mean pairwise distance between positively selected sites and a null distribution generated from randomly drawn surface (solvent exposed) sites (Figure 3B). Comparing the observed mean to the null distribution (10,000 permutations) lends statistical support to the hypothesis that these positively selected sites are clustered on the surface of KLK2 (*p* = 0.0043). The spatial distribution of selected sites on KLK2 provides an example of how positive selection can lead to inferences about evolution of protein function. In this case, a change in substrate is suggested.

**Figure 4:**
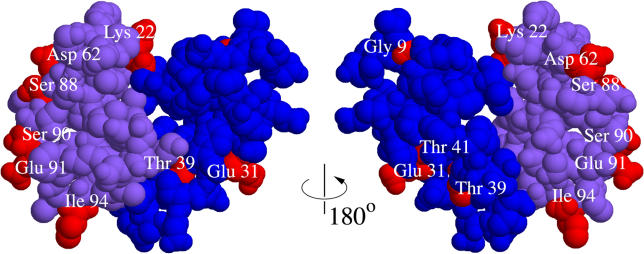
Positively Selected Sites on MSMB are Spread across the Protein Surface. According to sites models of codon evolution, 42% of MSMB residues experienced adaptive pressure to alter their amino acids. Those predicted with high support are shown in red on this threaded structural model of human MSMB. Blue and purple residues demarcate two structural domains of the protein [62]. The amino acid sites show no clustering and are almost significantly dispersed throughout the protein (*p* = 0.066). This pattern is quite different from that shown by KLK2 (Figure 3). Although MSMB is one of the most abundant human seminal proteins, its function remains unknown.

MSMB is one of the most abundant proteins in human seminal plasma, yet its function remains unknown. It is also evolving rapidly, with an estimated 42% of codons under positive selection (Table 1). Positively selected sites are found all over the exterior of a threaded structure of MSMB (Figure 4), in contrast to the clustering seen on KLK2. When a clustering test is performed on MSMB selected sites, the observed mean distance falls just short of being significantly dispersed (*p* = 0.066). This dispersed pattern suggests that selection has acted uniformly on the surface of MSMB and no distinct functional regions can be inferred.

Because few sites could be mapped onto structural models of transmembrane serine protease 2 (TMPRSS2), ACPP, and acyl-coA-binding protein (DBI), spatial distributions were less distinct, and clustering was not seen; however, some functional hypotheses may be made. Selected sites were predicted in two domains of TMPRSS2, the serine protease and the low-density lipoprotein receptor. When selected sites in the protease domain are mapped onto a threaded three-dimensional structure, they all occupy exterior positions on the same face, opposite of the protease active site. No interactions are confirmed in this region, but TMPRSS2 is thought to be activated through cleavage at a site located on this face [31]. The selective pressure on ACPP may be related to its substrate; a selected surface site (V77) neighbors two active site residues (R79 and H257) in the solved crystal structure [32]. Although this selected site was only moderately supported in Bayes Empirical Bayes analysis (*p* = 0.824), it is intriguing because of its proximity to the active site in an otherwise conserved region.

Structural analysis of selected sites and biochemical characterization are complementary approaches for elucidating the biological roles of these proteins. For example, our evidence of selection in KLK2 implies a testable change in substrate binding during primate evolution. As more coding sequences are determined, prediction of selected sites will improve, allowing site-specific selective pressures to be evaluated in functional contexts.

### Loss of Function

Evidence for loss of function in several species was seen for two candidate genes, *TGM4* and *KLK2*. Interestingly, both of these genes are involved primate semen coagulation. Prostate-specific TGM4 forms semen coagulum and copulatory plugs through cross-linking by its transglutaminase (TG) domain. Sequence from gorilla showed a homozygous, 11-basepair deletion in exon 7 at the start of the TG domain, a frameshift that would lead to early termination at amino acid 293 of 684. This deletion is likely fixed in gorilla populations, since four additional gorillas showed the same homozygous deletion. Abrogation of transglutaminase activity is likely since this exon contains ~20% of the TG domain and the remaining 80% falls downstream. Similarly, the sequenced *Hylobates lar* individual was homozygous for an early stop at codon position 411 downstream of the TG domain and before the first transglutaminase C-terminal domain.

In *KLK2,* the *Macaca mulatta* individual showed a homozygous change altering the active site residue D120 to alanine, which would eliminate proteolytic activity. This change was not seen in the four other Old World monkeys examined, including *Macaca nigra*. This suggests that abrogation of KLK2 activity occurred in those macaques closely related to *M. mulatta* or in that species alone.

Other evidence suggests loss of the *KLK2* gene from gorilla and lesser apes. Although *KLK2* sequence was obtained in several divergent New and Old World monkeys, only the first and last exons (1 and 5) were obtained from gorilla, despite several amplification conditions and primer combinations. When conditions were relaxed, PCR products from exons 2 through 4 of the paralog *PSA* were obtained instead, suggesting that *KLK2* was lost in the gorilla lineage. Similar difficulties were encountered in all three analyzed species of genus *Hylobates,* suggesting a similar loss in lesser apes. *PSA* and *KLK2* are paralogous genomic neighbors and likely arose through tandem duplication, so that unequal crossing-over could lead to deletion of one of the paralogs.

## Discussion

Several seminal fluid proteins show dynamic evolutionary histories, significant positive selection, and variable selective pressure between lineages. Multiple instances of loss of function also hint at changing selective pressure. Seminal protein adaptation could result from several potential pressures, including sexual selection, pathogen response, and coevolution with changing binding partners and substrates. It is hypothesized that sexual selection, namely sperm competition and sexual conflict, is a major driving force behind the adaptive evolution of *Drosophila* seminal fluid proteins [33], and could be responsible for primate divergence as well.

### Copulatory Plug Candidate Genes

In some primate species, the degree of semen coagulation is high enough to form a firm copulatory plug, a mechanism of sperm competition. Four prostate-specific candidate genes *(TGM4, KLK2, PSA,* and *ACPP)* participate in formation or dissolution of human seminal coagulum [34,35]. Significant positive selection is seen in *TGM4, KLK2,* and *ACPP,* along with significant variation in selective pressure between lineages for *TGM4, ACPP,* and *PSA*. Additionally, both of the genes showing loss of function participate in the formation *(TGM4)* or dissolution *(KLK2)* of semen coagulum. Loss of function of gorilla *TGM4* is consistent with the lack of semen coagulation in gorilla [12] and with past evidence of early stop codons in alleles of gorilla *semenogelins I* and *II* [9,10]*.* Degeneration of semen coagulation may also be occurring in the lar gibbon, as its *TGM4* coding sequence shows an early stop codon. Loss of semen coagulation is consistent with the mating systems of gorillas and gibbons, since both species are considered monoandrous, so males are not competitive postmating.

After a copulatory plug has been set, breaking it down is a strategy for competing males to win fertilizations. Positive selection seen in *ACPP* and *KLK2* could be due to optimization of this function. KLK2 proteolytically activates PSA, a protease that breaks down semen coagulum. The likely loss of function of *KLK2* observed in the rhesus macaque, gorilla, and lesser apes could result in reduced ability to dissolve semen coagulum. This change could reflect either lack of constraint for this function or adaptive value.

### Conflict over Sperm Levels

Human seminal fluid factors, such as prostaglandin E, can locally suppress female immune response [36]. This function may be related to conflict between males and females over sperm levels. As sperm competition leads to higher sperm levels, chances of polyspermy increase, causing females to limit sperm numbers and strengthen barriers to fertilization. Candidate genes *TGM4* and *MSMB* could serve to protect sperm from immune attack in the reproductive tract; evidence suggests that they both bind to sperm surfaces. TGM4 may deter attack by altering the sperm surface [37,38], and MSMB was found to be the main immunoglobulin binding factor in human seminal plasma [39,40]. Hence, these proteins may play roles in suppressing immune response against sperm, resulting in positive selection. Although highly expressed in human prostate, MSMB is also found in other mucous tissues [41], so a role in general pathogen defense must also be considered.

### Pathogen Response

Like other secretions, seminal fluid contains protective antipathogenic factors. One candidate, *PIP,* has a likely role in host defense. *PIP* shows strong signs of positive selection, with 25% of codons estimated at a high *d*_N_/*d*_S_ of 7.56. Notably, when just apes and one Old World monkey are analyzed, *PIP* shows highly significant positive selection (*p* = 0.00024). This secreted aspartyl proteinase is expressed at high levels in prostate and other exocrine glands. It is thought to play a role in host defense by binding bacteria, and it may suppress T-cell apoptosis [42,43]. Protection of sperm and the male reproductive tract from pathogens may also drive divergence of other seminal fluid proteins.

### Antagonistic Pleiotropy

The major source of seminal fluid proteins is the prostate, a common site of male cancer. Disease research may benefit from studies of selection, since positive selection is often associated with human disease genes. In an analysis of 7,645 genes, those showing signs of positive selection were overrepresented in genes associated with disease in the Online Mendelian Inheritance in Man catalog [44]. In addition, Nielsen et al. found several genes involved in tumor suppression and apoptosis among those showing the strongest signs of positive selection between human and chimpanzee [45]. Also, the cancer susceptibility genes *BRCA1* and *angiogenin* show signs of positive selection [46–49]. Such selection could result in antagonistic pleiotropy, a phenomenon in which adaptation in one respect brings deleterious effects in another. It is important to explore the possibility that adaptive evolution of seminal fluid factors contributes to disease through pleiotropic effects. Adaptation in prostate-expressed genes may benefit primates during their reproductive lifespan, but could lead to damaging side effects in later life.

### Screen Utility

Overall, this human-chimpanzee selective pressure screen was successful in identifying seminal fluid genes with significant signs of positive selection. This is notable because the screen compared two closely related species with relatively few nucleotide differences per gene. Since seven of eight candidate genes showed statistically significant positive selection, we expect a fraction of other genes with elevated pairwise *d*_N_/*d*_S_ ratios to be under positive selection.

### Conclusion

The lower limit for primate seminal fluid proteins under positive selection is nine, from seven proteins in this study plus *semenogelins I* and *II*. Given the rate of support in this study, we speculate that there are others from the set of screened genes, as well as from genes not included in this screen. In conclusion, primate seminal fluid contains several proteins that exhibit dynamic evolutionary histories involving positive selection and loss of function. Extensive adaptive evolution in seminal proteins may be common to internally fertilizing taxa, since evidence of positive selection is seen in both *Drosophila* and primates.

## Materials and Methods

### Selective pressure screen.

A list of 161 proteins identified in human seminal fluid was compiled from mass spectrometry studies of seminal plasma and prostasomes [20,21]. Prostate-expressed genes were identified from an expression study of whole normal prostate (NCI CGAP Pr22) from the Prostate Expression Database (http://www.pedb.org) [22]. Of 4,277 unique ESTs from the study, 2,858 were traced to unique accession numbers in the reference sequence (RefSeq) database. Human exons encoding these seminal fluid proteins and prostate-expressed genes were retrieved from the UCSC Table Browser (http://genome.ucsc.edu/cgi-bin/hgTables). Each human exon was aligned to the best BLASTN hit over a threshold of 1 × 10^−10^ from chimpanzee whole genome shotgun contigs [50], and coding sequence alignments were created for evolutionary analysis.

Pairwise values of *d*_N_/*d*_S_ for each human-chimpanzee coding sequence alignment were estimated by CODEML of the PAML package [17]. It was noted that some perceived substitutions resulted from poor-quality chimpanzee sequence, so substitution base calls in all gene candidates were manually verified in raw sequence chromatograms found in the sequence reads database [http://www.ncbi.nlm.nih.gov/Traces/trace.cgi/]). Presence of a signal sequence was predicted using SignalP [51]. Statistical significance of the difference between secreted versus nonsecreted *d*_N_/*d*_S_ values was evaluated by a permutation test comparing the differences between average *d*_N_/*d*_S_ values for random subsets through 100,000 permutations.

### Sequencing of candidate genes.

Coding portions of nine genes were sequenced in divergent primates. Total DNA from the following primates were obtained from the Coriell Institute for Medical Research (Camden, New Jersey, United States): *Pan troglodytes, P. paniscus, Gorilla gorilla, Pongo pygmaeus abelii, Macaca mulatta, M. nemestrina, Erythrocebus patas, Saguinus labiatus,* and *Ateles geoffroyi*. DNA samples from the following species were obtained through the Integrated Primate Biomaterials and Information Resource (IPBIR; http://www.ipbir.org/ ): *Hylobates gabriellae, H. lar, H. syndactylus, Cercopithecus cephus,* and *Papio anubis.* Gorilla DNA samples were a generous gift from Evan Eichler at the University of Washington, Seattle, Washington, United States. Sequences of the following genes were obtained from GenBank: *M. fascicularis PSA, Papio hamadryas MSMB*, *Saguinus oedipus MSMB,* and *M. fuscata PIP*. Human coding sequences were taken from RefSeq entries in the UCSC genome browser (http://genome.ucsc.edu). Lemur *KLK2* exons were retrieved from a BAC clone (GenBank accession number AC153325) sequenced by the NIH Intramural Sequencing Center (http://www.nisc.nih.gov/).

PCR was used to amplify exon-containing fragments from total DNA of various primates. PCR primers were designed from human introns, and clade-specific primers were designed when possible. PCR conditions and primer sequences are available from the authors upon request. Single-band PCR products were sequenced using Big Dye v.3.1 (Applied Biosystems, Foster City, California, United States). Sequence analysis was done using Phred, Phrap, and Consed [52,53]. High-quality sequence was used to generate coding sequences for each species based on human splice sites. Splice acceptor and donor sites were systematically checked for preservation of GT and AG nucleotides. Multiple alignments were made for each gene using ClustalW [54]. The close relationship between primates allowed for confident multiple alignments with few gaps. For estimation of *d*_N_/*d*_S_ at sites or lineages, we removed secretion signal sequences and those species sequences showing loss of function.

### Evolutionary analysis.

Phylogenetic relationships between the studied primates were taken from published studies [55–57]. Pairwise differences in *d*_N_ and *d*_S_ were calculated by MEGA version 3.0 [23], using a modified Nei-Gojobori (Jukes-Cantor) codon model with standard error computed analytically. Maximum likelihood evolutionary analysis was done with CODEML of the PAML 3.14 package [17], which estimates parameters for codon models of evolution. In order to ensure correct estimation of model parameters, we checked for convergence by running the optimization multiple times with different starting values of the omega parameter. The omega parameter estimates the *d*_N_/*d*_S_ ratio and is used to determine selective pressure on codon sites, where a value greater than one is indicative of positive selection. Statistical significance is determined by a likelihood ratio test comparing a neutral model, where omega is limited to the interval (0, 1), to a selection model with an additional class of codons whose omega value is allowed to be greater than one. Different codon models were used for testing variation in *d*_N_/*d*_S_ between sites; the models were compared as follows: (neutral to selection) M1 to M2, M7 to M8, and M8A to M8. All three comparisons gave similar results; we report those for M8A to M8 in Table 1. Model M1 (neutral) allows two classes of codons, one with omega over the interval (0,1) and the other with an omega value of one. Model M2 (selection) is similar to M1 except that it allows an additional class of codons with a freely estimated omega value. Model M7 (neutral) estimates omega with a beta-distribution over the interval (0, 1), while model M8 (selection) adds parameters to M7 for an additional class of codons with a freely estimated omega value. M8A (neutral) is a special case of M8 that fixes the additional codon class at an omega value of one [58]. Significance of positive selection found among codon sites was estimated both with and without the sequences from the initial screen (human and chimp). Such exclusion avoids a bias of selecting lineages from the screen with high numbers of nonsynonymous substitutions. When significant signs of positive selection were found, specific codon sites subjected to positive selection were predicted using a Bayes Empirical Bayes approach employed in CODEML [28]. Such an approach gives more reliable probability calculations than past methods, since it takes into account sampling errors in estimates of model parameters. To evaluate variation in selective pressure over a phylogeny, the branch model of CODEML estimated *d*_N_/*d*_S_ values for each branch. The branch model is compared to the null hypothesis, model M0, in which all lineages have the same *d*_N_/*d*_S_ value.

### Structural analysis.

Threaded protein structures for KLK2, DBI, TMPRSS2, and MSMB were created with SwissModel using human primary sequence [59]. Structural analysis of *ACPP* sites was done on a solved crystal structure [32]. Protein structure images were produced using RasMol 2.7.2.1 [60].

Statistical significance of spatial clustering of amino acids was assessed by comparing the mean pairwise physical distance between positively selected sites to the mean distance between an equal number of random surface sites. A *p*-value was obtained by making this comparison 10,000 times. Surface sites were defined as those amino acids that are at least 20% solvent-exposed over their surface area. Solvent exposure was calculated using GETAREA 1.1 [61].

## Supporting Information

Table S1 Selective Pressure Screen Comparing 161 Human and Chimpanzee Seminal Protein Genes This table shows pairwise *d*_N_/*d*_S_ estimates from two different methods.
(79 KB XLS)

### Accession Numbers

The GenBank (http://www.ncbi.nlm.nih.gov/) accession numbers of the genes discussed in this paper are *M. fascicularis PSA* (AY647976), *Papio hamadryas MSMB* (U49786), *Saguinus oedipus MSMB* (AJ010154, AJ010155, AJ010158), and *M. fuscata PIP* (AB098481).

The sequences generated in this study have been submitted to GenBank under accession numbers DQ150438 through DQ150526.

## Abbreviations

TG: transglutaminase
